# Structure-Based Modeling of Sigma 1 Receptor Interactions with Ligands and Cholesterol and Implications for Its Biological Function

**DOI:** 10.3390/ijms241612980

**Published:** 2023-08-19

**Authors:** Meewhi Kim, Ilya Bezprozvanny

**Affiliations:** 1 Department of Physiology, UT Southwestern Medical Center at Dallas, Dallas, TX 75390, USA; 2 Laboratory of Molecular Neurodegeneration, Peter the Great St Petersburg State Polytechnical University, 195251 St. Petersburg, Russia

**Keywords:** sigma 1 receptor, crystal structure, modeling, biophysics, neurodegenerative disease

## Abstract

The sigma 1 receptor (S1R) is a 223-amino-acid-long transmembrane endoplasmic reticulum (ER) protein. The S1R plays an important role in neuronal health and it is an established therapeutic target for neurodegenerative and neuropsychiatric disorders. Despite its importance in physiology and disease, the biological function of S1R is poorly understood. To gain insight into the biological and signaling functions of S1R, we took advantage of recently reported crystal structures of human and *Xenopus* S1Rs and performed structural modeling of S1R interactions with ligands and cholesterol in the presence of the membrane. By combining bioinformatics analysis of S1R sequence and structural modelling approaches, we proposed a model that suggests that S1R may exist in two distinct conformations—“dynamic monomer” (DM) and “anchored monomer” (AM). We further propose that equilibrium between AM and DM conformations of S1R is essential for its biological function in cells, with AM conformation facilitating the oligomerization of S1R and DM conformation facilitating deoligomerization. Consistent with experimental evidence, our hypothesis predicts that increased levels of membrane cholesterol and S1R antagonists should promote the oligomeric state of S1R, but S1R agonists and pathogenic mutations should promote its deoligomerization. Obtained results provide mechanistic insights into signaling functions of S1R in cells, and the proposed model may help to explain neuroprotective effects of S1R modulators.

## 1. Introduction

The sigma-1 receptor (S1R) is a 223-amino-acid-long transmembrane protein residing in the endoplasmic reticulum (ER) [1,2,3]. The S1R is expressed at high levels in the nervous system [4,5,6]. Juvenile Amyotrophic Lateral Sclerosis 16 (ALS16) is caused by a homozygous E102Q mutation in the SIGMAR1 gene [7]. The additional loss of function mutations in the S1R causes distal hereditary motor neuropathies (dHMN) [8,9,10,11,12]. Furthermore, some variants of the S1R gene are associated with increased risk for Alzheimer’s disease (AD) [13]. These genetic findings support the important role of S1R in neuronal health. S1R activation by agonists has resulted in neuroprotective effects in multiple cellular and animal models of neurodegeneration [2,3,14,15,16,17]. S1R is also a target for drugs of abuse, including cocaine [18].

Despite its importance in physiology and disease, the biological function of S1R is poorly understood [2,3,17]. It appears that S1R is able to modulate multiple cell biological and physiological processes in neurons, but the exact mechanism of its actions is not clear. Recently, our laboratory proposed a hypothesis that the biological function of S1R in cells is mediated by its ability to form cholesterol-enriched microdomains in ER, in particular in mitochondria-associated membranes (MAMs) and other ER contact sites [16,19,20]. This hypothesis was based on the identification of a consensus cholesterol-binding motif in S1R sequence and a series of biophysical and cell biological experiments. It was also consistent with previous observations of S1R association with cholesterol in biochemical experiments [21,22,23]. To evaluate this idea further and to better understand biological functions of S1R in cells, we here performed a series of structural modeling experiments by taking advantage of recently solved crystal structures of human and *Xenopus* S1R [24,25,26]. In our modeling studies, we assumed that the N-terminal of S1R is cytosolic and the C-terminal is located in the ER lumen, in agreement with most cell biological studies and APEX-labeling electron microscopy experiments [27]. Based on the obtained results, we propose that the biological function of S1R depends on the transition between well-folded and rigid “anchored monomer” conformation and more disordered “dynamic monomer” conformation. Moreover, we propose that equilibrium between these two conformations of S1R is affected by agonists, antagonists, the presence of cholesterol in the membrane and pathogenic mutations in the S1R sequence. S1R is a therapeutic target for treatment for a variety of neurodegenerative disorders, and the model proposed in this study may help to explain the neuroprotective effects of S1R modulators.

## 2. Results

### 2.1. Co-Existence of Two Conformations of Sigma 1 Receptor BIND Domain

**Figure 1:**
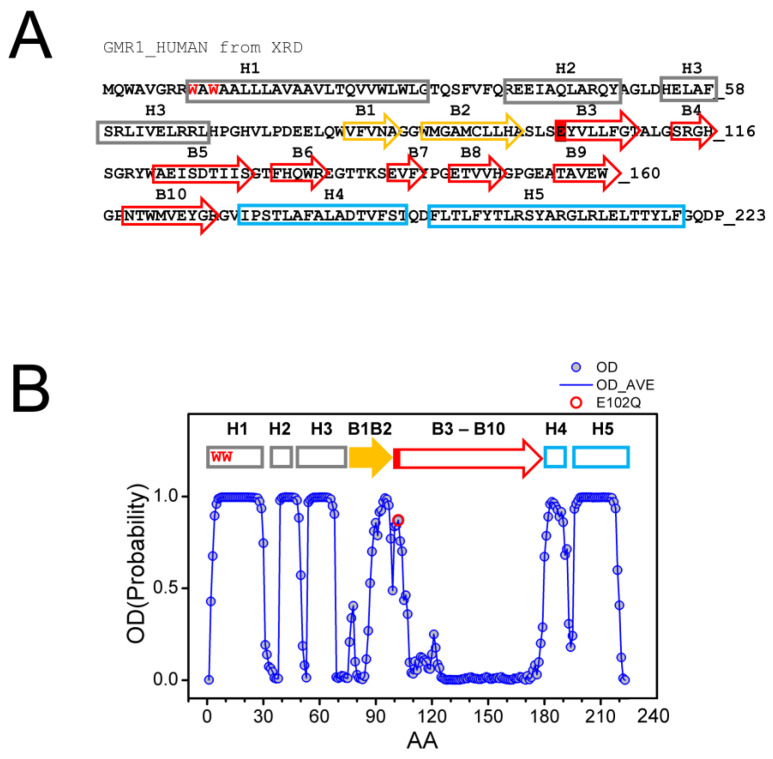
Primary sequence and secondary structure elements of S1R. (**A**) Complete primary sequence of human S1R (hS1R) is shown. Secondary structure elements—H1–H5 α helices and B1–B10 β-strands are labeled based on 5HK1 structural file (hS1R bound to PD144418) [24]. Alpha helices in crystal structure are labeled in grey, B1-B2 β strands are labeled in yellow, and B3-B10 β-strands are labeled in red. W9 and W11 residues predicted to form cholesterol binding site [20] are shown with a red font. The position of E102 residue mutated in ALS16 [7] is labeled red. (**B**) Results of PSIPRED analysis of hS1R primary sequence. Ordered probability (OD) is plotted for each amino acid (open blue circles and line). Secondary structure elements from the crystal structure [24] are shown above the OD plot. Position of E102 residue mutated in ALS16 [7] is shown by a red circle.

The structure of Sigma 1 receptor (S1R) was recently solved using X-ray diffraction (XRD) crystallography in lipid cubic phase for human S1R (hS1R) [24,25] and with the sitting drop method for *Xenopus* S1R (xS1R) [26] as a single transmembrane domain protein. The receptor has five α helices, including one transmembrane domain, and ten β strands, which make up the ligand binding domain (BIND). The secondary structure elements are labeled on the sequence of the human Sigma 1 receptor based on the crystal structure [24] (Figure 1A). The structure of S1R solved by XRD has significant differences from the structures predicted for S1R based on the previous NMR and biochemical labeling studies [28] (discussed in [3]). Such differences may potentially be explained by the co-existence of multiple conformations of S1R, with one of these conformations stabilized by XRD crystallization conditions and other, less ordered conformations present in NMR experiments. To evaluate this possibility, we analyzed the primary sequence of human S1R with PSIPRED [29] and calculated the probability of ordered conformation (OD) for each amino acid. Based on this analysis, we discovered that there is a good agreement between calculated OD values and XRD in the amino-terminal and carboxy-terminal regions on S1R (Figure 1B). Indeed, the amino acids that make up α-helices H1–H3, α-helices H4/H5 and β-strands B1/B2 were predicted with a high probability to be present within a folded domain. In contrast, amino acids in the middle portion of the protein that correspond to β-strands B3–B10 are predicted to be intrinsically disordered by PSIPRED (Figure 1B). This is a region of S1R that forms the ligand-binding BIND region. Based on this analysis, we proposed that the corresponding region of S1R (E102–G176) may adopt two different conformations—well folded conformation, as observed by XRD [24,25,26], and partially unfolded or disordered conformation. The hypothesis that the BIND region adopts disordered conformation in solution is consistent with a significant drop in ^15^N chemical shift observed in this region by NMR studies of S1R in solution [28]. We further proposed that the binding of the ligand stabilizes the folded conformation of the BIND region, as observed in crystal structures of hS1R [24,25] and xS1R [26].

### 2.2. Effects of Association with Cholesterol on Sigma 1 Receptor H1 α-Helix Conformation

**Figure 2:**
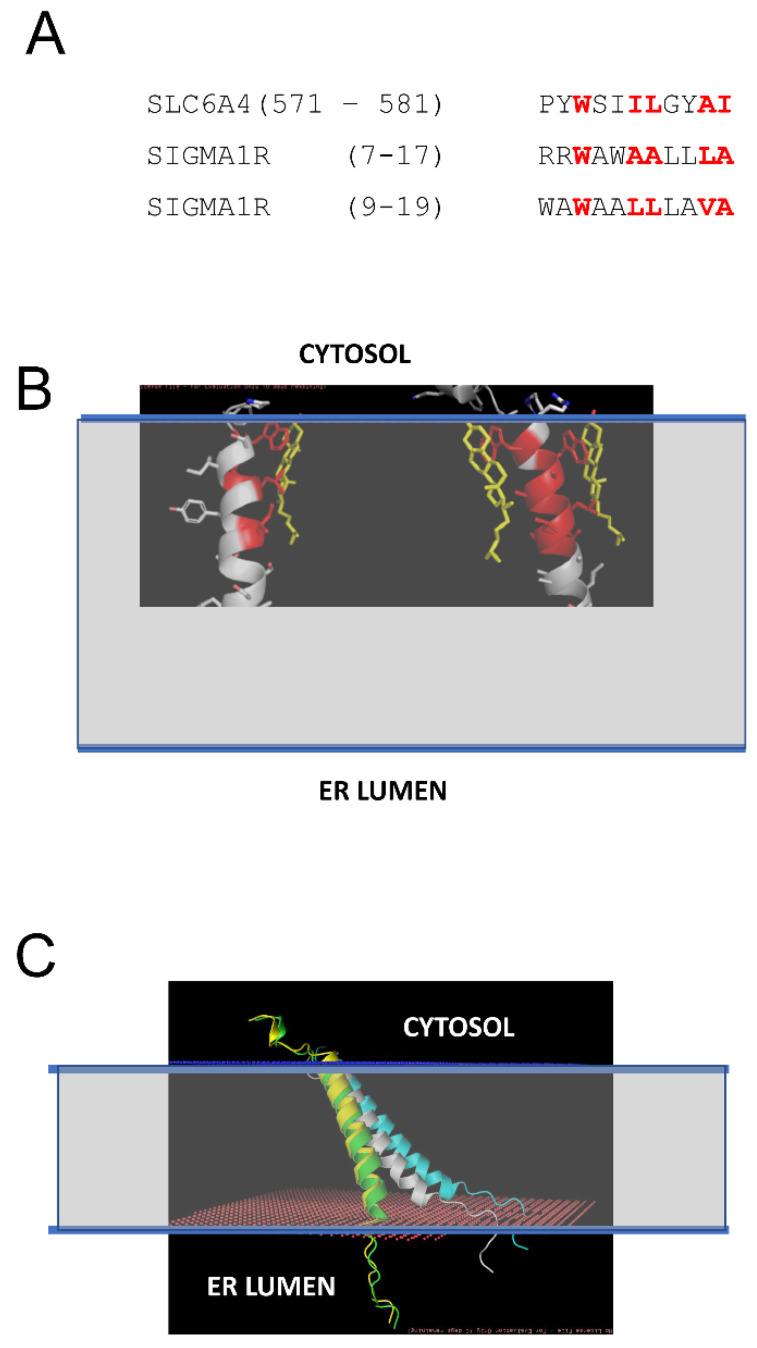
Structural model of S1R association with cholesterol in the membrane. (**A**) Sequence alignment of the cholesterol-binding site from SLC6A4 [36] and tandem putative cholesterol-binding sites (CARC motifs) from S1R [20]. The residues directly involved in interactions with cholesterol in SLC6A4 and predicted to interact with cholesterol on S1R are colored red. (**B**) Structural models of S1R with one or two cholesterol molecules bound. The residues directly involved in interactions with cholesterol in the models are colored red. Cholesterol molecules are shown by yellow. The predicted membrane boundaries are shown by the blue lines and the transparent space between the lines. (**C**) Anchored models of H1 α-helix from S1R. The models are shown for S1R in the absence of cholesterol (white), with one CHO bound (green), with two CHO bound (yellow), and for WWLL mutant (blue). The predicted membrane boundaries are shown by the blue lines and the transparent space between the lines.

Previous studies demonstrated that S1R can directly interact with cholesterol and ceramides in mitochondria-associated membranes (MAMs) [21,22,23]. In our recent study, we identified tandem CARC-like motifs between amino acids 4 and 17 of S1R and demonstrated that mutating two critical tryptophanes (W9 and W11) in this motif abolishes S1R clustering in the presence of cholesterol [20]. CARC motifs have been suggested to predict cholesterol-binding locations in proteins [30,31,32,33], but the predictive value of these motifs has also been challenged [34]. To further validate and confirm our discovery of the cholesterol-binding motif in S1R, we performed structural modeling of the S1R complex with cholesterol. To achieve this, the PDB databank was searched for known structures of proteins in complexes with cholesterol (CHO). Such a search identified 35 PDB files in the database, and within these files 11 structures of transmembrane helixes directly bound to cholesterol were manually identified. These 11 structures were used for structural alignment with the structure of the S1R transmembrane domain from 6DK0 [25] using the CCP4 Gesamt package [35]. This alignment identified structures of serotonin transporter SLC6A4 (5I6X) [36] and CB1 cannabinoid receptor CNR1 (5XRA) [37] as the closest structural analogs for the S1R transmembrane domain. Sequence alignment indicated a strong similarity between the SLC6A4 cholesterol-binding sequence (aa 571–581) and two putative overlapping cholesterol binding sites in the S1R sequence (aa 7–17 and aa 9–19) (Figure 2A). Structural modeling performed using Coot [38] based on the S1R transmembrane domain structure from 6DKO [25] resulted in a model with one or two cholesterol molecules interacting with S1R via positions W9 and W11 in the H1 α-helix of S1R (Figure 2B: cholesterol molecules are colored in yellow, cholesterol-interacting residues in S1R are colored in red). The critical role of W9 and W11 residues in the association of S1R with cholesterol is consistent with our previous conclusions based on sequence analysis and mutagenesis experiments [20].

We generated S1R H1 α-helix structural models for CHO-free (WT, white), single-CHO-associated (CHO1, green), and two-CHO-associated (CHO2, yellow) forms (Figure 2C). We also generated a model for the W9L/W11L double mutant of the S1R H1 α-helix (WWLL, blue) that was investigated in the our previous experiments [20] (Figure 2C). We noticed that for CHO-free and WWLL forms, the S1R H1 α-helix with the tilted angle in the membrane is most energetically favorable, whereas single- and double-CHO-associated forms of the S1R H1 α-helix were embedded vertically through the membrane to maximize the energy of association with vertically positioned cholesterol molecules (Figure 2C). To evaluate the effects of association with cholesterol, we calculated the energy of membrane association (E_M_) for the H1 α-helix in different conditions. The calculated E_M_ for the S1R H1 α-helix was ~45 kJ in the absence of cholesterol. We determined that in the presence of cholesterol in the membrane, the energy of hydrophilic interactions decreases, and the energy of hydrophobic interactions increases for the H1 α-helix. As a result, association with cholesterol compensates the energetic cost of the re-arrangement of the H1 α-helix of S1R from “tilted” to “vertical” conformation without a significant change in the total energy of membrane association—the calculated change in E_M_ (dE_M_) was close to 0 for both CHO1 and CHO2 forms when compared to cholesterol-free form. In contrast, the WWLL mutant energy of both hydrophobic and hydrophilic interactions increased, with the total dE_M_ equal to 5 kJ for the WWLL form and an even more “tilted” conformation of the H1 α-helix in the membrane.

Based on this analysis, we concluded that the S1R H1 α-helix may exist in two different conformations. One conformation is perpendicular to the membrane and stabilized by association with cholesterol (Figure 2C, yellow and green), similar to conformation observed in the XRD structures of human S1R solved in lipid cubic phase [24,25]. Another conformation is a tilted conformation that is adopted by the S1R H1 α-helix in the absence of cholesterol (Figure 2C, white). Consistent with this prediction, somewhat tilted conformation of the S1R H1 α-helix was observed in more recent XRD structure of *Xenopus* S1R solved in solution by the sitting drop method [26]. We further conclude that the W9L/W11L double mutant of S1R H1 α-helix adopts even more tilted conformation in the membrane independent of the presence or absence of cholesterol (Figure 2C, blue).

### 2.3. Effects of Pathogenic Mutations on Sigma 1 Receptor Association with the Membrane

**Figure 3:**
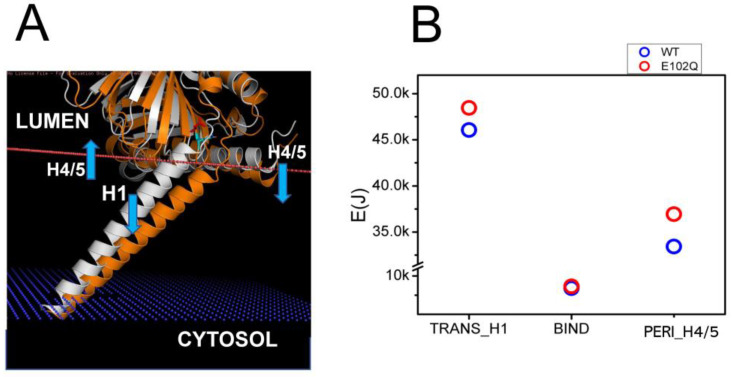
Structural model of E102Q effects on S1R association with the membrane. (**A**) Structural models of wild type hS1R (white) and hS1R-E102Q mutant (orange) are compared. The membrane boundary is shown by the orange line. The blue arrows indicate the displacement of H4/5 and H1 α-helices in hS1R-E102Q mutant structure. (**B**) E_M_ calculations for H1, BIND and H4/5 domains for wild type hS1R (blue circles) and hS1R-E102Q mutant (red circles).

Juvenile Amyotrophic Lateral Sclerosis 16 (ALS16) is caused by a homozygous E102Q mutation in the SIGMAR1 gene [7]. Previous biophysical studies have indicated that E102Q mutation disrupts the higher-order oligomerization of the S1R by affecting its conformation [39]. Consistent with this prediction, in our previous studies we observed the mislocalization of the S1R-E102Q mutant in cells [20]. In order to understand the potential effects of E102Q mutation on the conformation of S1R, we compared structural models of wild type S1R and S1R-E102Q embedded in the membrane (Figure 3A). We noticed that E102Q mutation resulted in the movement of the H1 α-helix (Figure 3A, orange) into the membrane, increasing its tilt even further. The H4/H5 α-helices in the S1R-E102Q mutant were tilted into the perimembrane region of the membrane (Figure 3A). Based on XRD analysis, the S1R domain structure consists of three domains which are involved in different membrane associations—H1 transmembrane (TM) domain, H4/5 permembrane (PM) domain and BIND domain in ER lumen that does not interact with the membrane [24,25,26]. Through energetic calculations, we determined that as a result of the E102Q mutation the energy of the S1R membrane association was increased for the H1 α-helix by 2.39 kJ and for H4/5 α-helices by 3.51 kJ, without a significant change in the energy of membrane association for the BIND region (Figure 3B). From these results, we concluded that the E102Q mutation is likely to stabilize S1R in a “tilted” conformation of the H1 α-helix by increasing the total energy of S1R association with the membrane by 5.9 kJ due to a change in the position of the H4/H5 α-helices.

### 2.4. Effects of Ligand Association on Sigma 1 Receptor Conformation

In the previous studies, we determined that S1R agonists prevent the formation of S1R clusters or promote the disassembly of S1R clusters in the cholesterol-containing membranes and that these effects can be blocked by S1R antagonists [20]. These conclusions are in agreement with most previous biochemical and biophysical studies of S1R [40,41,42], although the opposite conclusion was reached by some groups [43]. To obtain better information on conformational changes induced by agonist and antagonist association with S1R, we compared the AlphaFold-predicted structure of hS1R in ligand-free form and the crystal structures of human S1R obtained in the presence of antagonists (PD144418, 4-IBP, NE-100, haloperidol) [24,25] and agonist (+)-pentazocine [25]. We also compared the crystal structures of *Xenopus* S1R in complex with antagonist S1RA, agonist PRE084 and in the apo form [26].

**Figure 4:**
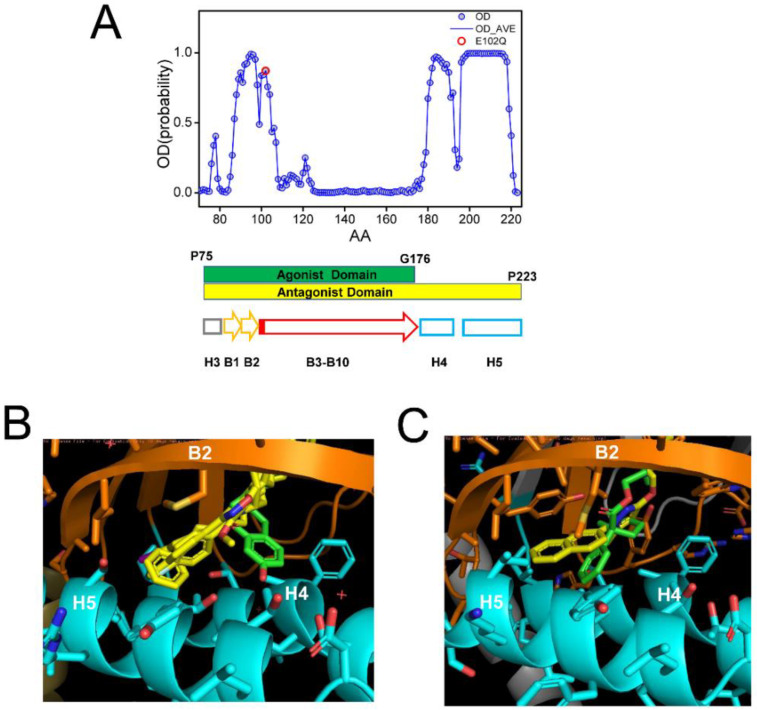
Analysis of ligand-binding site of S1R. (**A**) Results of PSIPRED analysis of a portion of hS1R primary sequence that includes the ligand-binding site (P75–P223). Ordered probability (OD) is plotted for each amino acid (open blue circles and line). Secondary structure elements from the crystal structure [24] shown for the corresponding region of S1R below the OD plot. Position of E102 residue mutated in ALS16 [7] is shown by a red circle. The boundaries of regions involved in interaction with agonist (green) and antagonists (yellow) are shown by bars below the OD plot. (**B**) Overlay of fragments of the hS1R structures with 4 antagonists (all shown in yellow) from 5HK1 (hS1R bound to PD144418) [24], 5HK2 (hS1R receptor bound to 4-IBP) [24], 6DK0 (hS1R bound to NE-100) [25], 6DJZ (hS1R bound to haloperidol) [25] and agonist (shown in green) 6DK1 (hS1R bound to (+)-pentazocine) [25]. (**C**) Overlay of fragments of the xS1R structures with antagonist (shown in yellow) 7W2F (xS1R bound to PRE084) [26] and agonist (shown in green) 7W2D (xS1R bound to S1RA) [26]. On panels B and C, B2 β-strand is shown in orange and H4/H5 α-helices are shown in blue.

In all crystal structures of S1R complexes with agonists and antagonists, the ligand-binding cavity is highly occluded [24,25,26], leaving an open question for the path of ligand entry and exit to the binding pocket. Molecular dynamics simulations indicated that there are two potential paths for the ligands to enter the S1R ligand binding pocket—PATH1 and PATH2 [44]. The PATH1 pathway involves the unfolding and refolding of the cupin-fold β-barrel body of the BIND domain as the ligand enters the S1R ligand binding pocket [44]. A similar hypothesis has been previously proposed based on the analysis of the crystal structure of human S1R [24,25]. The PATH2 pathway involves the temporary movement of H4/5 α-helices, which opens the entry path for the ligands to enter the S1R ligand binding pocket from the side of the membrane [44]. It has been suggested that PATH2 is more likely based on the crystal structure of *Xenopus* S1R in the “open conformation” [26]. The main argument against the PATH1 hypothesis was that it is not energetically favorable as it requires the unfolding of the BIND domain β-barrel [26]. However, as discussed already, a large portion of the BIND region (B3–B10 β-strands, E102–G176) is predicted to be disordered (Figure 4A), and our hypothesis is that in the absence of the ligand the BIND domain of S1R exists in a partially or completely unfolded state, so there are no energetic costs involved in breaking down the β-barrel body. Following the entry of the ligand, the S1R BIND domain folds around it, resulting in a high affinity association, as observed in crystal structures [24,25,26]. These ideas are more aligned with the PATH1 ligand entry hypothesis. Although the structure of the xS1R apo-form was also solved in the folded conformation of BIND domain, this is most likely because the disordered conformation of the BIND domain is incompatible with crystallization conditions and only the folded conformation of BIND was selected or induced during crystallization.

It was stated In the previous crystal structure reports that agonists and antagonists interact with the S1R ligand binding pocket in a similar way [25,26]. However, when we compared structures of agonist and antagonist complexes with hS1R (Figure 4B) and xS1R (Figure 4C), we noticed significant differences at the atomic level. The overlaid crystal structures of the human S1R ligand binding pocket complexed with PD144418, 4-IBP, NE-100, or haloperidol (antagonists, yellow) [24,25] and (+)pentazocine (agonist, green) [25] are shown in Figure 4B. The overlaid crystal structures of the *Xenopus* S1R ligand binding pocket complexed with S1RA (antagonist, yellow) [26] and PRE084 (agonist, green) [26] are shown in Figure 4C. As previously reported [24,25,26], both agonists and antagonists are able to stabilize their association with S1R via electrostatic interaction between basic nitrogen in the ligands and the carboxy group in E172 (hS1R) (Figure 4B) or E169 (xS1R) (Figure 4C). In addition to this electrostatic interaction, antagonists (yellow) are able to stabilize their position via a series of hydrophobic interactions with B2 β-strand and H4 and H5 α-helices (Figure 4B,C). For hS1R, the residues involved in these hydrophobic interactions are M93, L95, A98, I178, L182 and A185 (Figure 4B). Analogous residues are involved in hydrophobic interactions with antagonist in xS1R (Figure 4C). In contrast, agonists (green) are not able to establish these hydrophobic interactions with residues in the B2 β-strand and H4 or H5 α-helices due to the presence of Hydroxybenzene in the structure (Figure 4B,C). As a result, the agonist binding region (P75-G176) is shorter than the antagonist binding region (P75–P223) in hS1R (Figure 4A). The G176 residue is within a low OD region (Figure 4A), suggesting that even the presence of the agonist BIND domain may have a tendency to unfold and adopt an intrinsically disordered structure. Thus, our conclusion is that antagonists will stabilize the folded conformation of the BIND domain due to additional interactions between the ligand and H4/5 α-helices, whereas the agonist will destabilize it and promote the partial unfolding of the BIND domain due to the presence of an extra Hydroxybenzene ring within the binding pocket (Figure 4B,C). Perhaps for this reason, crystal structures of hS1R in complex with multiple agonists (PD144418, 4-IBP, NE-100, haloperidol) have been solved, but only the structure with a single agonist (+)pentazocine has been obtained so far [24,25]. In additional modeling studies, we established that E102Q mutations had minimal effects on the structure of the S1R complexed with the NE-100 antagonist in the AM conformation (Supplementary Figure S1). This is in contrast to significant changes in the DM conformation of the apoform of S1R resulting from the same mutation (Figure 3).

## 3. Discussion

### 3.1. Dynamic Equilibrium of Two Conformations of Sigma 1 Receptor

**Figure 5:**
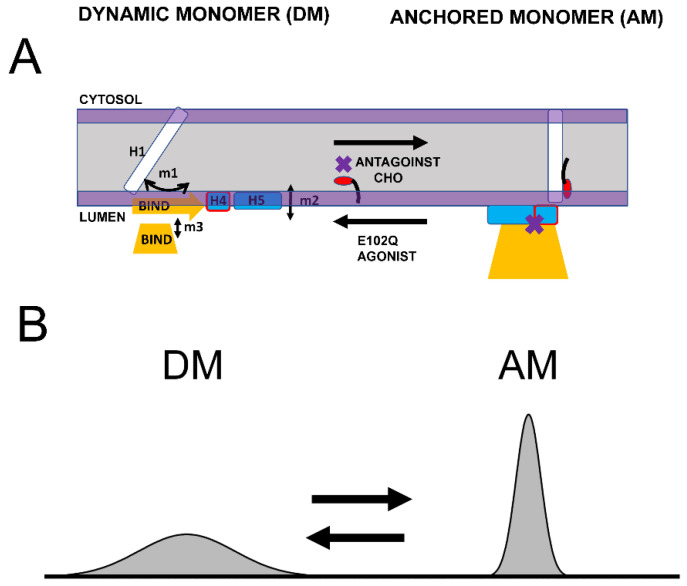
Proposed conformational equilibrium of S1R. (**A**) Schematic depiction of dynamic monomer (DM) conformation and anchored monomer (AM) conformations of S1R. Possible movements of H1 α-helix (m1), H4/5 α-helices (m2) and unfolding of BIND domain (m3) are shown. The equilibrium between DM and AM conformations is affected by S1R agonists, S1R antagonists, cholesterol (CHO) in the membrane and E102Q pathogenic mutation, as shown. (**B**) Proposed distribution of different S1R conformations. AM conformation is well defined and forms a narrow peak in conformational space. DM conformation is a spectrum of molecular species that differ from each other in the tilt of H1 α-helix, position of H4/5 α-helices relative to the surface of the membrane and the folding state of BIND domain. As a result, DM conformation forms a broad peak in conformational space.

Based on the structural modeling of S1R domains in this study, we would like to propose the co-existence of two conformations of S1R—the “dynamic monomer” (DM) conformation and “anchored monomer” (AM) conformation (Figure 5A). There are three key differences between these conformations—(1) H1 α-helix is perpendicular to the membrane in the AM conformation but tilted in DM conformations (m1 movement); (2) H4 and H5 α helices are located on the membrane surface in the AM conformation and in the perimembrane space in the DM conformation (m2 movement); and (3) the BIND domain is folded to cupin-fold β-barrel body in the AM conformation but partially or completely unfolded in the DM conformation (m3 movement). The AM conformation of S1R is the conformation that is observed in all known crystal structures for S1R [24,25,26]. Due to its intrinsic flexibility, the DM conformation is not compatible with crystallization conditions and has not been directly observed. But the existence of such a conformation is indirectly supported by ^15^N chemical shift data in NMR studies of S1R in solution [28] and by the ability of the ligands to enter a binding pocket that is completely occluded in all crystal structures [44]. In contrast to the well-defined AM structure, we also propose that DM conformation exists as a continuum of different conformations of S1R (Figure 5B). The spectrum of DM conformations spans different degrees of H1 α-helix tilt (m1 movement), different positions of H4 and H5 α helices relative to the surface of the membrane (m2 movement), and different degrees of unfolding of the BIND domain (m3 movement).

We suggest that in cells both conformations of S1R exist in dynamic equilibrium, with some receptors adopting a well-defined AM form and some receptors present in conformations within the “DM conformational spectrum” (Figure 5B). Our analysis predicts that the association of cholesterol with the H1 α-helix or the binding of S1R antagonists to the BIND domain shifts the equilibrium towards the AM state (Figure 5A). In contrast, pathogenic mutation E102Q shifts the equilibrium towards the DM state (Figure 5A). Similarly, the binding of S1R agonists to the BIND domain appears to be destabilized by steric interference from the Hydroxybenzene ring, shifting the equilibrium towards the DM state (Figure 5A).

### 3.2. Implications for Sigma 1 Receptor Biological Function

**Figure 6:**
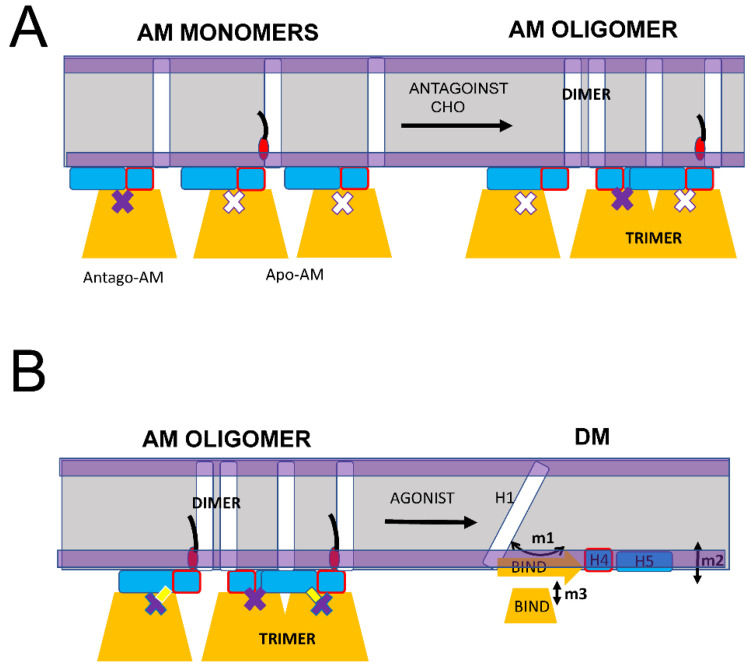
Proposed mechanism of S1R oligomerization. (**A**) S1R in the AM monomeric form promotes the generation of large oligomers due to the formation of BIND-BIND trimers and H1-H1 dimers. Large oligomers are stabilized in the presence of cholesterol (CHO) or S1R antagonists. (**B**) S1R agonists cause the conversion of AM conformation of S1R to DM conformation resulting in a loss of BIND-BIND and H1-H1 intramolecular associations and the disassembly of large S1R oligomers and clusters.

As we previously proposed, the main biological function of S1R in cells appears to be the formation and maintenance of cholesterol-enriched microdomains in the ER membrane [16,19,20]. Moreover, in our recent review article [16], we proposed that neuroprotective effects of S1R agonists can be explained by their ability to release a “reserve pool” of plasma membrane and secreted proteins, which accumulate in these microdomains. To perform this function, S1R needs to form large-order oligomers. Consistent with this idea, large oligomeric species that include 100 or more S1R molecules were observed in our single molecule in vitro imaging experiments [20] and in biochemical and cell biological studies [24,41,43,45,46]. We would like to propose that the AM conformation of S1R is able to form large order oligomers due to the ability of S1R monomers to form trimers via the association of BIND domains and dimers via association with H1 α-helices (Figure 6A). Both types of these intermolecular interactions between AM forms of S1R have been observed in crystal structures [24]. This model predicts that the formation and growth of S1R oligomers should be enhanced in the presence of cholesterol or S1R antagonists (Figure 6A), in agreement with experimental observations [20,41,46]. The model also predicts that the formation of S1R oligomers should be impaired by pathogenic E102Q mutation, also consistent with the known properties of this mutant [20,39]. As discussed above (Figure 5), the binding of the agonist is expected to destabilize the AM conformation of S1R, which is expected to result in the conversion of DM conformation and the disassembly of high-order oligomeric structures (Figure 6B). Indeed, the disassembly of S1R clusters in response to the agonist has been observed experimentally [20,40,41,42].

In conclusion, we propose that transitions between the DM spectrum of conformations and the AM conformation of S1R forms the basis for its biological function in cells. While in AM form, S1R is able to form large-scale oligomers via H1-H1 and BIND-BIND intramolecular interactions (Figure 6A). Conditions that stabilize AM conformation, such as a high local concentration of cholesterol or the presence of S1R antagonists, are expected to facilitate the formation of large-scale oligomers of S1R. In contrast, conditions that destabilize AM conformation and shift the equilibrium towards the DM spectrum of conformations, such as E102Q pathogenic mutation or the presence of S1R agonists, are expected to prevent the formation or lead to the disassembly of large-scale oligomers of S1R. S1R is a therapeutic target for treatment for a variety of neurodegenerative disorders, and the proposed model (Figure 5 and Figure 6) may help to explain the neuroprotective effects of a variety of S1R ligands and modulators which appear to be directly linked to their ability to modulate S1R and cholesterol clustering in the ER membrane [16].

## 4. Materials and Methods

### 4.1. Secondary Structure Analysis

To investigate the secondary structures of S1R protein, PDB files of human S1R (hS1R) and *Xenopus* S1R (xS1R) structures determined with X-ray diffraction (XRD) were obtained from the databank—5HK1 (hS1R bound to PD144418) [24], 5HK2 (hS1R bound to 4-IBP) [24], 6DK0 (hS1R bound to NE-100) [25], 6DJZ (hS1R bound to haloperidol) [25], 6DK1 (hS1R bound to (+)-pentazocine) [25], 7W2F (xS1R bound to PRE084) [26] and 7W2D (xS1R bound to S1RA) [26]. The structure of hS1R in apo form was obtained from Alphafold as coordinated file AF-Q99720-F1-V4 [47,48] and compared to the apo-form structure of xS1R (7W2E from [26]). For analysis of the ligand binding crystal structures, hS1R bound with antagonists (5HK1,5HK2,6DK0,6DJZ) and agonist (6DK1) were overlayed in PyMOL (https://pymol.org/) to identify the differences. The crystal structures of xS1R bound with antagonist S1RA (7W2D) and agonist PRE084 (7W2F) were also overlayed in PyMOL (https://pymol.org/) to identify the differences. The crystal structures with antagonists and agonist were also compared with AlphaFold-predicted structure of apo form (AF-Q99720-F1-V4) and with apo form structure of xS1R (7W2E) by aligning these structures using the CCCP4I2 Gesamt package [35].

S1R protein sequence (Q99720) and mutation file from Uniprot were used for secondary structure prediction by PSIPRED (http://bioinf.cs.ucl.ac.uk/psipred/, accessed on 13 February 2023) [29] and the mutants model was applied to obtain the membrane-associated PDB files using MEMEMBED (http://bioinf.cs.ucl.ac.uk/psipred/, accessed on 13 February 2023) [49]. E102Q mutation was introduced to the structure of hS1R bound to NE-100 (6DK0) by Coot and the MEMEMBED program was used to predict the energy of membrane association, resulting in the predicted model.

For modeling association with cholesterol (CHO), the hS1R transmembrane domain was extracted from 6DK0 [25]. The PDB databank was searched for known structures of proteins in complex with cholesterol. This search identified 35 PDB files in the database, and within these files 11 structures of transmembrane helixes directly bound to cholesterol were manually identified. These 11 structures were used for structural alignment with the structure of the S1R transmembrane domain using the CCP4 Gesamt package [35]. This alignment identified structures of serotonin transporter SLC6A4 (5I6X) [36] and CB1 cannabinoid receptor CNR1 (5XRA) [37] as the closest structural analogs for the S1R transmembrane domain. Sequence alignment indicated a strong similarity with SLC6A4. The membrane association models of S1R H1α-helix without CHO, with one CHO and with two CHO molecules and S1R-WWLL were obtained by using MBEDED.

### 4.2. The Calculation of the Membrane-Association Energy of S1R

The S1R structure predicted by Alphafold AF-Q99720-F1-V4 was used for energetic calculations. The structure was divided into three domains for membrane association energy (E_M_) calculations—transmembrane H1 α-helix (Trans_H1), peri-membrane H4/H5 α-helix (Peri_H4/5) and ligand binding domains composed on B1-B10 β-strands (BIND). The E_M_ was calculated separately for each domain in wild type hS1R, and hS1R-E102Q mutant and added up to obtain total E_M_ value. The membrane association (E_M_) of H1 and H4/5 was calculated using MODA [50] and MEMEMBED [49]. The E_M_ was calculated separately for Hydrophobic (Pho) and Hydrophilic (Phil) membrane association energies. Pho was defined as an interaction with acyl groups of lipids and Phil was defined as interaction with solvent/perimembrane area. The interaction energy for each domain was obtained by integrating the residual energy by MODA for the Phil and Pho interactions within a domain.

## Data Availability

Not applicable.

## Supplementary Materials

The supporting information can be downloaded at: https://www.mdpi.com/article/10.3390/ijms241612980/s1.
