# Supplementary material for: Structure-Based Modeling of Sigma 1 Receptor Interactions with Ligands and Cholesterol and Implications for Its Biological Function
Source: Int J Mol Sci. 2023 Aug 19;24(16):12980. doi: 10.3390/ijms241612980 (PMC10455549; doi:10.3390/ijms241612980)
Supplement: Supplementary file 1 [file ijms-24-12980-s001.zip › ijms-2553097-supplementary.pdf]

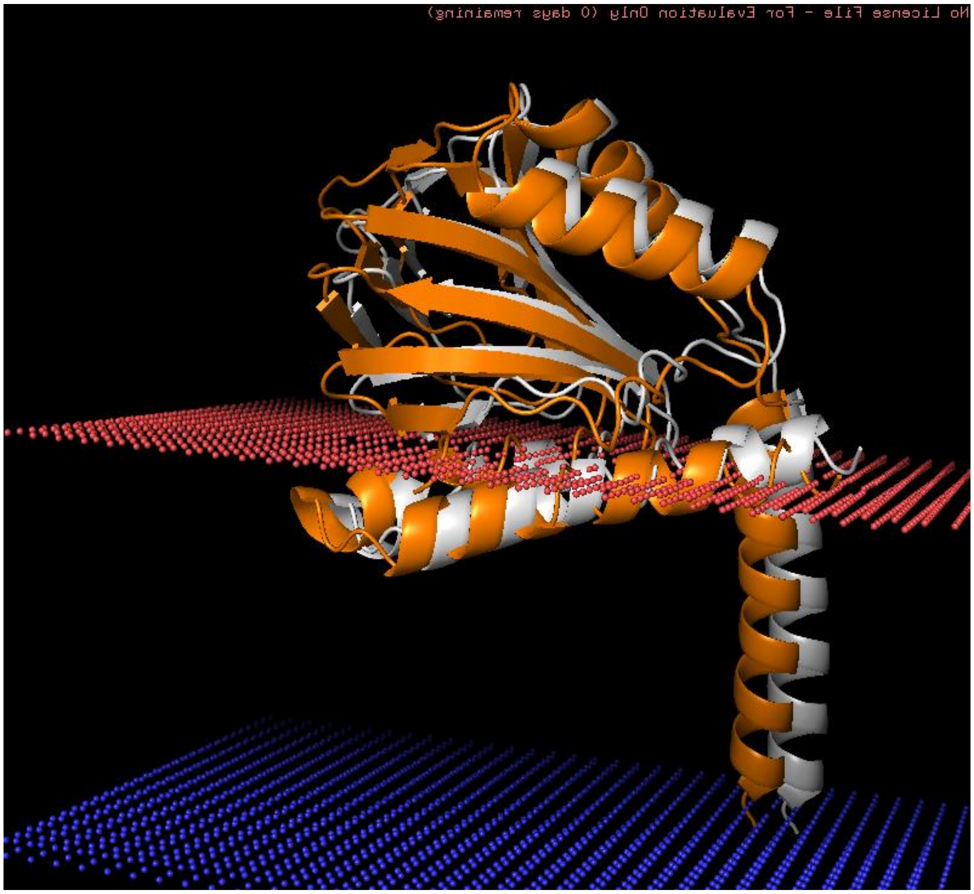

Supplementary Figure S1. Structural model of E102Q effects on S1R association with the membrane in the presence of bound NE-100.

Structure of wild type hS1R bound to NE100 (white) from PDB file 6DK0. E102Q mutation was introduced by Coot and resulting effects on membrane association was calculated by EMBEDED. Predicted conformation of hS1R-E102Q in complex with NE-100 is shown (orange).
